# A conceptually new treatment approach for relapsed glioblastoma: Coordinated undermining of survival paths with nine repurposed drugs (CUSP9) by the International Initiative for Accelerated Improvement of Glioblastoma Care

**DOI:** 10.18632/oncotarget.969

**Published:** 2013-04-13

**Authors:** Richard E. Kast, John A. Boockvar, Ansgar Brüning, Francesco Cappello, Wen-Wei Chang, Boris Cvek, Q. Ping Dou, Alfonso Duenas-Gonzalez, Thomas Efferth, Daniele Focosi, Seyed H. Ghaffari, Georg Karpel-Massler, Kirsi Ketola, Alireza Khoshnevisan, Daniel Keizman, Nicolas Magné, Christine Marosi, Kerrie McDonald, Miguel Muñoz, Nathan David Sassoon, Mohammad H. Pourgholami, Iacopo Sardi, Avishay Sella, Kalkunte S. Srivenugopal, Marco Tuccori, Weiguang Wang, Christian R. Wirtz, Marc-Eric Halatsch

**Affiliations:** ^1^ IIAIGC Headquarters, Dean of Studies, Burlington, VT, USA; ^2^ Weill Cornell Medical College, NY, USA; ^3^ University of München, München, Germany; ^4^ University of Palermo, Palermo, Italy; ^5^ Chung Shan Medical University Hospital, Taichung, Taiwan; ^6^ Palacky University, Olomouc, Czech Republic; ^7^ Wayne State University, Detroit, USA; ^8^ Instituto de Investigaciones Biomedicas UNAM, Instituto Nacional de Cancerología, Mexico City, Mexico; ^9^ Johannes Gutenberg University, Mainz, Germany; ^10^ University of Pisa, Pisa, Italy; ^11^ Tehran University of Medical Sciences, Tehran, Iran; ^12^ University of Ulm, Ulm, Germany; ^13^ University of British Columbia, Vancouver, Canada; ^14^ Shariati Hospital, Tehran University of Medical Sciences, Tehran, Iran; ^15^ Oncology Department, Meir Medical Center, Tel Aviv University, Israel; ^16^ Institut de Cancérologie Lucien Neuwirth, Saint-Priest en Jarez, France; ^17^ Medical University of Vienna, Wein, Austria; ^18^ University of New South Wales, Sydney, Australia; ^19^ Virgen del Rocío University Hospital, Sevilla, Spain; ^20^ Texas Tech University Health Sciences Center, Amarillo, USA; ^21^ Meyer Children's Hospital, Firenze, Italy; ^22^ Assaf Harofeh Medical Center, Zerifin, Israel; ^23^ University of Wolverhampton, Wolverhampton, UK

**Keywords:** angiotensin, aprepitant, artesunate, auranofin, captopril, cytokines, disulfiram, glioblastoma, ketoconazole, nelfinavir, neurokinin, sertraline, temozolomide

## Abstract

To improve prognosis in recurrent glioblastoma we developed a treatment protocol based on a combination of drugs not traditionally thought of as cytotoxic chemotherapy agents but that have a robust history of being well-tolerated and are already marketed and used for other non-cancer indications. Focus was on adding drugs which met these criteria: a) were pharmacologically well characterized, b) had low likelihood of adding to patient side effect burden, c) had evidence for interfering with a recognized, well-characterized growth promoting element of glioblastoma, and d) were coordinated, as an ensemble had reasonable likelihood of concerted activity against key biological features of glioblastoma growth. We found nine drugs meeting these criteria and propose adding them to continuous low dose temozolomide, a currently accepted treatment for relapsed glioblastoma, in patients with recurrent disease after primary treatment with the Stupp Protocol. The nine adjuvant drug regimen, Coordinated Undermining of Survival Paths, CUSP9, then are aprepitant, artesunate, auranofin, captopril, copper gluconate, disulfiram, ketoconazole, nelfinavir, sertraline, to be added to continuous low dose temozolomide. We discuss each drug in turn and the specific rationale for use- how each drug is expected to retard glioblastoma growth and undermine glioblastoma's compensatory mechanisms engaged during temozolomide treatment. The risks of pharmacological interactions and why we believe this drug mix will increase both quality of life and overall survival are reviewed.

Preamble: ecce turtur. ipse proficit tantum con collum foras.

## I INTRODUCTION

In the effort to improve the current prognosis in glioblastoma of one or two years' survival after primary diagnosis even with the best of treatments, we have created a new adjuvant approach, termed CUSP9, presented here. As of early 2013, standard initial treatment consists of temozolomide and irradiation after maximal primary resection, typically referred to as the “Stupp Protocol” [1, 2]. There is no standard or agreed upon single best approach to recurrent glioblastoma after this initial treatment. A total of 22 clinical trials reported in 2012, all using various new, recurrent glioblastoma treatments that all had sound established activity in pre-clinical study [1, 3-23]. Yet none reported more than minimal quality of life (QOL) or overall survival (OS) benefit. Many were stopped early for increased morbidity, drastically decreased QOL, or for meeting pre-established futility criteria.

Dörner et al. recently reported that even after placement of carmustine (BCNU) wafers along the surgical cavity wall, the recurrence pattern remains largely local with meager meaningful advantages to patients in OS or QOL increases [24]. We present here a position paper from The International Initiative for Accelerated Improvement of Glioblastoma Care on a treatment plan aiming for more lasting tumor control while not adding to patients' side effect burdens. Our treatment plan- termed CUSP9- aims to increase QOL and OS compared to current recurrent glioblastoma treatments by adding nine already-marketed growth factor-inhibiting drugs to low dose continuous temozolomide.

We review here the rationale for this nine drug mix using already-marketed drugs, as adjuvants to improve effectiveness and tolerability of low dose continuous temozolomide in treatment of glioblastoma at first post-Stupp protocol recurrence. The total ten drugs of CUSP9 are in three function categories:

- Established use in recurrent glioblastoma-temozolomide,
- Sound pre-clinical evidence supporting potential benefit based on documented inhibition of growth factors or a pathogenic driver- aprepitant, artesunate, auranofin, disulfiram with copper gluconate, nelfinavir,
- Published reports of increased OS with use but of uncertain significance and drugs with less robust theoretical support- captopril, sertraline, ketoconazole

As many growth enhancing systems have been identified in glioblastoma, and many currently-marketed drugs not traditionally thought of as cytotoxic have shown to have inhibitory activity at one or another of these systems, we searched the literature to find such drug- glioblastoma growth factor matches that would be unlikely to add to patient side effect burden based on our experience of long use of the respective drug in humans for non-cancer indications. Drugs were selected also on the basis of having little potential for seriously aversive effect or interaction with each other and, as an ensemble, had reasonable likelihood of concerted and coordinated activity against key biological features of glioblastoma growth. In the Conclusion section, we outline why CUSP9 can be expected to improve QOL as well versus current simpler treatments.

Pharmacologic risks of each drug individually and permutations of all possible combinations were carefully evaluated and presented in section IV. CUSP9 PHARMACOLOGY. The only potential for harmful interaction that we can foresee is the potential interaction between artesunate and auranofin. We believe this risk can be managed by slow up titration of doses and frequent monitoring as described in section V. Partial CUSP's, Risk Reduction, Risk Assessment. Otherwise the risks of these drugs used together was assessed to be low with the understanding that unexpected interactions may occur and this risk will require exceptional vigilance, as with any new treatment but particularly so in a regimen of this complexity.

There is now broad recognition that multiple cross-covering growth promoting signaling paths and cell death avoiding mechanisms are active in glioblastoma [25- 30]. As Siegelin et al. stated in referring to glioblastoma, “Drug discovery for complex and heterogeneous tumors now aims at dismantling global networks of disease maintenance” [29]. Or as formulated by Eyler et al. also referring to glioblastoma, these “networks of disease maintenance” require commensurate corresponding efforts directed to understanding “the exact nature by which many of the pathogenic drivers connect” [30]. On this basis we generate CUSP9. The conclusion of recognition of many global networks and multiple pathogenic drivers is the requirement for many pathogenic drivers' inhibitors as CUSP9 provides.

Multiple pathogenic drivers and interconnected growth promoting paths of glioblastoma maintenance are concepts reminiscent of a well-known problem in medicine, expressed as a metaphor- “The Three Locks Problem”. A door with three locks will not open any better if one or two of the locks are unlocked. Likewise blocking one or two growth factors may not result in any slowing of glioblastoma growth due to alternate paths that take over for the blocked one[s]. How do we proceed if glioblastoma has twenty locks on it? In logic terms “if A then not B” does not imply “if not A then B” [if growth path “A” remains active then glioblastoma won't be stopped (“not B”) does not imply that if growth path “A” is effectively inhibited then glioblastoma will be stopped (“B”)].

**Figure 1:**
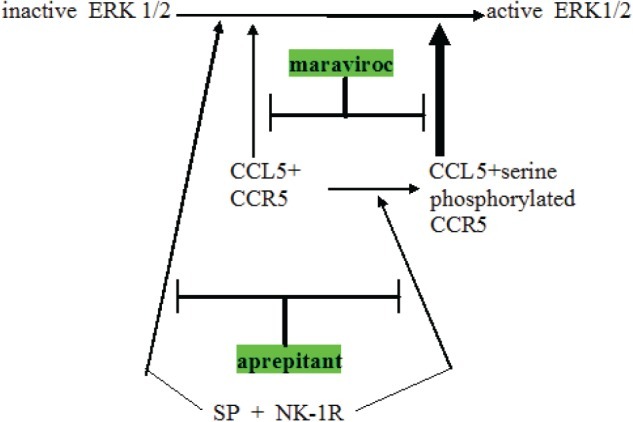
Schema showing relationship between CCR5 and neurokinin receptor (NK-1R) signaling operative in glioblastoma Note three points: (a) NK-1R signaling can augment CCR5 signaling by converting less active plain CCR5 to the more active serine phosphorylated CCR5, (b) NK-1R and CCR5 can cross-cover for each other, both independently can activating ERK1 / 2 and (c) expected synergy between aprepitant and maraviroc in blocking this aspect of glioblastoma growth promotion. Maraviroc is a newly approved blocking drug at the CCR5 cytokine receptor.

## II RATIONALE

### II.1 Established use in recurrent glioblastoma-temozolomide

Temozolomide is a 194 Da alkylating cytotoxic cancer chemotherapy drug. Current standard primary glioblastoma treatment, the Stupp Protocol, is, with minor variations, temozolomide and irradiation to tumor area after maximal resection that spares areas vital to good QOL [1, 2, 31, 32]. There is no generally accepted standard treatment for glioblastoma that has recurred after Stupp Protocol treatment. Our plan is to give temozolomide 50 mg/m^2^ daily without pause (or until toxicity) at first recurrence after a completed Stupp Protocol. Multiple other first recurrence treatments have been reported but none have exceeded the QOL maintenance and a median OS of 30 to 41 weeks that this regimen provides [2, 33, 34].

### II.2 Sound pre-clinical evidence supporting potential benefit based on documented growth factor or a pathogenic driver's inhibition- aprepitant, artesunate, auranofin, disulfiram, nelfinavir

#### II.2.1 aprepitant

Aprepitant is a 534 Da oral neurokinin-1 receptor (NK-1R) antagonist approved for use in treating chemotherapy induced nausea and vomiting (CINV), an indication for which it is quite safe and effective [35, 36], even during highly emetogenic regimes [36-40]. Substance P is an eleven amino acid signaling neuropeptide that belongs to the tachykinin family of peptides. Substance P is the natural ligand of NK-1R that by binding to NK-1R, in addition to generating CINV, forms a regulatory link in many biological functions in cancer such as proliferation, angiogenesis, migration, and metastasis.

**Table 1:** Summary of CUSP9, listing the drugs with a short unreferenced description of the rationale or expected advantage accruing from its use References are given in the text.

DRUG	EXPECTED BENEFIT
aprepitant	Nausea reduction, inhibit growth by blocking NK-1R
artesunate	Increase ROS, empirical anti-glioma effects, survivin inhibition
sertraline	Empirical longer OS, improved mood, documented anti-proliferation effect in glioma cells
captopril	Empirical longer OS, MMP-2 & MMP-9 inhibition, prevents AT-2 stimulation, lowers IL-18 stimulated VEGF, TNF, & IL-8
auranofin	Thioredoxin reductase inhibition, cathepsin B inhibition, increased i.c. ROS, empirical [& potentially dangerous] synergy with artesunate,
nelfinavir	HSP90 inhibition, MMP-2 & MMP-9 inhibition, decreased signaling at multiple receptors, i.a. TGF-beta, increased i.c. ROS, decreased AKT activation, lower VEGF, IL-8, ICE inhibition
temozolomide	A common & accepted treatment for recurrent glioblastoma
disulfiram	ALDH inhibition, glutathione inhibition, increase ROS, lowers IL-18 stimulated VEGF, TNF, & IL-8, MMP-2 & MMP-9 inhibition, proteosome inhibition, SOD inhibition, P-glycoprotein inactivation, MGMT inhibition.
Cu gluconate	Adequate Cu may be a requirement for disulfiram activity
ketoconazole	Drug efflux inhibitor at BBB, permits higher brain ritonavir (or nelfinavir) concentrations, 5-lipoxygenase inhibitor, thromboxane synthase inhibitor, empirical anti-glioma effect

Muñoz et al. [41, 42] have been documenting in a series of articles the growth enhancing aspects of NK-1R signaling in several cancers over the last few years. They have been advocating trials of aprepitant in an anti-cancer role in these cancers. Since many cancers [43, 44], including glioblastoma [45] use Substance P signaling at NK-1R as a growth stimulating element it was logical enough to suggest aprepitant as treatment adjunct [46, 47]. An added benefit of aprepitant is the rarity of any side effect at all when used as treatment for CINV [37].

Empirically, Substance P stimulates glioma cells' proliferation [45]. NK-1R antagonists such as aprepitant, after binding to NK-1R, inhibit proliferation, have pro-apoptotic effects, and exert anti-angiogenic and anti-migration effects in pre-clinical models [42, 44, 48].

Aprepitant has central effects and good diffusion across the blood brain barrier (BBB). Moreover, it is eminently well tolerated [36, 37] even in a clinical trial as antidepressant using 300 mg/day. Side effects did not differentiate from placebo [49].

Considering the above data sets, plus the fact that glioma cells tend to overexpress NK-1R [50] and aprepitant shows a broad spectrum antitumor action, including in glioma models, it was natural to add aprepitant to CUSP9.

#### II.2.2 artesunate

Artesunate is a 384 Da orally available drug used in tablets alone or in fixed combinations with other drugs to treat malaria, particularly with drug-resistant Plasmodia strains, around the world and usually without a prescription [51]. Artesunate is one of several related semi-synthetic phytoderrived drugs, the artemisinin's, based on Artemisia annua, an herb used in Chinesse traditional medicine. In aggregate, worldwide artesunate consumption is massive.

As part of a screening campaign of the National Cancer Institute, USA, artesunate and other related artemisinin-type compounds were shown to have cytotoxicity towards 60 cell lines derived from 8 different tumor types, including CNS tumors [52]. That artesunate induces apoptosis in cancer cells was first shown by Efferth et al. in a leukemia cell line [53].

That there was no correlation between IC_50_ values of artesunate and mRNA expression of the multidrug resistance-conferring ABCB1 gene (coding for P-glycoprotein efflux pump) in the National Cancer Institute cancer cell lines and that artesunate is similarly active towards cell lines which over-expressing MDR1/P-glycoprotein [54-56] indicate that artesunate isn't a substrate for these chemotherapy defeating elements. Likewise, methotrexate-resistant cells with an amplified dihydrofolate reductase gene and hydroxyurea-resistant cells over-expressing ribonucleotide reductase were not cross-resistant to artesunate [56].

Particularly beneficial for glioblastoma treatment, the transfer of dihydroartemisinin, the first metabolite of artesunate, from plasma to lipid-rich brain structures is still increasing at a time when post-dose blood levels of both artesunate and dihydroartemisinin are decreasing in humans with malaria treated with artesunate [57].

**Figure 2:**
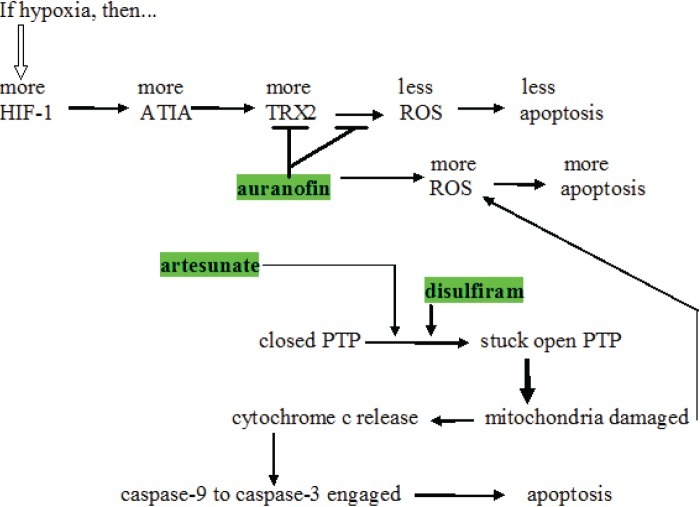
Schema showing several places where artesunate, auranofin, and disulfiram act to increase intracellular oxidative stress induced apoptosis protein; ROS = reactive oxygen species; TRX-2 = thioredoxin reductase; HIF-1 = hypoxia induced factor-1; ATIA = anti-TNF-alpha-induced apoptosis; PTP = mitochondrial outer membrane permeability transition pore.

A rough correlation of baseline antioxidant mRNA gene expression in the National Cancer Institute cancer cell lines with the IC_50_ values for artesunate indicated a role of reactive oxygen species (ROS) stress in artesunate's anti-cancer effect [58-60]. WEHI7.2 cells selected for resistance to hydrogen peroxide or transfected with thioredoxin (note strong thioredoxin reductase inhibition by another member of CUSP9, auranofin discussed below, see also Figs. 2. and 6.), manganese superoxide dismutase (that is inhibited by another CUSP9 member, disulfiram discussed below) or catalase were relatively artesunate-resistant compared to parental cells [58].

As tumor cells commonly contain more iron than corresponding normal cells, the question arises as to whether iron may be critical for artemisinin group medicines' action towards tumor cells. Cellular iron uptake and internalization are mediated in part by binding of transferrin-iron complexes to a transferrin receptor (CD71) on the cell surface membrane [61].

CD71 is highly expressed in tumors, including glioblastoma [62, 63]. CD71 expression was higher in a leukemia cell line and in U373 glioma tumor cells (48-95%) than in peripheral mononuclear blood cells of healthy donors (<2%) [64]. Iron and transferrin increased cytotoxicity of artesunate and artemisinin towards these cell lines [64].

Interestingly, exposure of artemisinin and its derivatives produced only marginal cytotoxicity to non-tumor cells [64, 65]. The growth of primary human fibroblasts was almost unaffected by artesunate concentrations up to 100 μM [64, 65]. The elegance of this approach of Efferth et al. is that it uses glioblastoma's hunger for growth as reflected in CD71 overexpression, an erstwhile growth enhancing attribute, to help kill these cells.

Artesunate also induces DNA breakage in a dose-dependent manner as shown by single-cell gel electrophoresis and confocal microscopy of the DNA double-strand break indicator gamma-H2AX [53, 66]. This is probably the proximate mode of damage leading to artesunate mediated cell death.

The epidermal growth factor receptor (EGFR, also known as HER-1) is an important target for cancer therapy and is particularly important specifically in glioblastoma pathophysiology [67-69]. Artesunate strongly enhanced erlotinib cytotoxicity to glioblastoma cells [70]. Artesunate enhanced cytotoxicity of doxorubicin in human acute T cell leukemia Jurkat cell line J16 and the human acute lymphoblastic leukemia cell lines CEM and Molt-4 [71]. Exposure of glioma cells to artesunate enhanced irradiation induced induction of apoptosis, G2/M arrest and DNA damage [72] and had similar effects in a non-small cell lung cancer cell line [73].

#### II.2.3 auranofin

Auranofin is a 678 Da drug approved to treat rheumatoid arthritis [74, 75]. Three sets of data suggest its use in adjuvant treatment of glioblastoma.

One of the probable mechanisms of action of auranofin in promoting cytotoxic drug cell death is given in Fig. 2. Anti-TNF-alpha-induced apoptosis protein (ATIA) is highly expressed in human glioblastoma [76]. ATIA knockdown in glioblastoma cells renders them sensitive to hypoxia-induced apoptosis. As shown in Fig. 2, ATIA up regulates thioredoxin reductase [76], a cytoprotective factor reducing intracellular ROS. As outlined below, it is precisely by inhibiting such cytoprotective thioredoxin reductase that we think auranofin exerts its primary anticancer effect. Strong thioredoxin reductase inhibition by auranofin is well-documented [77]. Mean circulating blood thioredoxin reductase levels were 31% higher in glioblaastoma patients compared to matched controls [78], indicating a significant pathophysiological role for thioredoxin reductase. Glioblastoma tissue levels of thioredoxin reductase were five times that of normal brain [78].

Shifting cancer cells' redox bias towards oxidizing, away from reducing is an active area of cancer research. As a major force in limiting oxidizing conditions within cells, thioredoxin reductase therefore becomes an attractive target in cancer treatment generally. Auranofin and other thioredoxin reductase inhibitors are in active research programs as adjunct to current cancer treatment [79, 80]. How thioredoxin reductase inhibition by auranofin shifts intracellular redox towards an oxidizing state is diagrammed in Fig. 6. Increased intracellular ROS generated by auranofin has been shown empirically [81, 82].

Multiple experimental thioredoxin reductase inhibitors other than auranofin are in various stages of development for cancer treatment after showing good pre-clinical activity [83, 84]. We intend to use the one we already have- auranofin.

As diagrammed in Fig. 6, NADP(H) is required for regeneration of reduced thioredoxin. Reduced thioredoxin is a major intracellular reducing agent. Glioma cell line proliferation remains unchanged as intracellular NADP(H) levels decrease until a level ~15% of normal is reached. At further decreases beyond that point proliferation and motility become progressively impaired and viability steeply declines as levels descend through <10% of normal [85].

We have empirical evidence of auranofin's cytotoxicity in multiple cancer models [86], for example ovarian cancer [87], head & neck cancer [88] and others.

Cathepsin B is an important element of glioblastoma cell invasion along Scherer's tracts [89, 90]. Two independent groups found that heavier cathepsin B immunohistochemical staining predicts shorter glioblastoma OS [91, 92]. Experimental reduction of cathepsin B inhibited glioblastoma growth and invasion in rodent glioblastoma models [93]. Auranofin inhibits cathepsin B and was previously suggested as a glioblastoma treatment adjunct on that basis [94].

#### II.2.4 disulfiram

Of all the ancillary drugs of CUSP9, disulfiram has the strongest evidence of potential benefit in glioblastoma. Disulfiram is a 297 Da oral aldehyde dehydrogenase (ALDH) inhibitor that results in accretion of acetaldehyde if ethanol is ingested. Such high levels of acetaldehyde are experienced as extremely unpleasant, hence alcohol consumption is discouraged, the current main clinical use of disulfiram. It has been in continuous use in the treatment of alcoholism since the 1940's [95].

After ingestion disulfiram is rapidly metabolized to diethyldithiocarbamate after binding copper in the stomach [96]. Disulfiram metabolism is complex [96] and the ultimate metabolic species relevant to ALDH inhibition or disulfiram's anti-cancer effects remain uncertain. In clinical use by far the most common observation is to see no side effects at all in those patients taking disulfiram 250 mg p.o. once or twice a day to treat alcoholism. An estimated 10% will experience an easily tolerated metallic taste at times.

Much of the seminal work on disulfiram's anti-cancer cell properties come from the lab of Weiguang Wang at the University of Wolverhampton [97-101]. Disulfiram's multiple anticancer properties have been demonstrated in pre-clinical models of breast, prostate, myeloma, leukemia, lung cancers, cervical adenocarcinoma, melanoma, neuroblastoma and colorectal cancer [102-112].

Disulfiram exerts significant anticancer activity in multiple contexts [112-118]. Particularly the stem cell sub-population of glioblastoma are ALDH positive [97, 98, 120-123], and susceptible to inhibition by disulfiram [97, 98, 100, 120-124]. We have increasing pre-clinical evidence that disulfiram, an old and inexpensive drug in continuous use for 6 decades in alcohol aversion therapy exerts significant anticancer activity [104, 112-118].

**Figure 3:** Grid of potential foreseeable interactions of CUSP9 drugs We recognize that unexpected interactions are not ruled out by this grid but the literature review behind this grid does make untoward reactions less likely. apr, aprepitant; sert, sertraline; cap, captorpil; Au, auranofin; NFV, nelfinavir; TMZ, temozolomide; DSF, disulfiram; cap, captopril; Cu, copper gluconate; ket, ketoconazole; art, artesunate; PK: potential pharmacokinetic interaction; PD: potential pharmacodynamic interaction

**Table:** 

sert	-								
cap	-	-							
Au	-	-	-						
NFV	PK	-	-	-					
TMZ	-	-	-	-	PD				
DSF	-	-	-	-	-	-			
Cu	-	-	-	-	-	-	-		
ket	PK	-	-	-	PD	PD	-	-	
art	-	-	-	PD	PK	-	-	-	PK
Drugs	apr	sert	cap	Au	NFV	TMZ	DSF	Cu	ket

Disulfiram has previously been documented to potentiate the effect of several chemotherapy agents in vitro: cisplatin [128], gemcitabine [101, 129] temozolomide [130], paclitaxel [100], docetaxel [131], cyclophosphamide [132], 5-fluorouracil [101, 102], doxorubicin [99], sunitinib [104], and BCNU [133]. Currently, disulfiram is in Phase I clinical trials in metastatic melanoma, in hormone refractory cancers with lung and liver metastases (www.%20clinicaltrials.gov, identifiers NCT00256230 and NCT00742911) as well as in prostate cancer (identifier: NCT01118741).

Dufour et al. randomized 64 women with non-metastatic high risk breast cancer in a double blind placebo controlled phase 3 clinical trial of adjuvant diethyldithiocarbamate vs. placebo [134]. Diethyldithiocarbamate 10 mg/kg p.o. or placebo was given once weekly for 9 months after the first chemotherapy cycle. After 6 years, overall survival was 81% in the diethyldithiocarbamate group vs. 55% in placebo group, a significant difference. Current recommended dosing of disulfiram during alcoholism treatment is 250-500 mg per day, equivalent to 3500 mg of diethyldithiocarbamate weekly compared to the ~700 mg of diethyldithiocarbamate weekly given in the Dufour et al. trial [134]. Even at 3000 mg p.o. per day disulfiram gave no serious adverse effects (135).

A detail-poor case report appeared in 1977 of a woman experiencing gradual remission of breast cancer metastases to spine, skull, pelvis and ribs after intermittent exposure to disulfiram and interspersed drinking bouts (136). She died from a fall after ten years of such intermittent, alternating (and overlapping?) alcohol-disulfiram exposure. Autopsy showed high blood alcohol level and microscopic nests of metastatic breast cancer in her bone marrow but no macroscopic disease [136].

A case report of a patient with metastatic ocular melanoma treated with disulfiram 250 mg/day p.o. and zinc gluconate 250mg/day p.o. showed decreasing tumor volume [110]. After 53 months of ongoing treatment with disulfiram (250 mg/d) and zinc gluconate (250 mg/d) this patient experienced no progression with no discernible drug side effects [110].

Suppression of breast cancer xenografts by disulfiram was shown in mice (137). Multiple in vitro models have recently demonstrated considerable anti-glioblastoma effects via proteasome and ALDH inhibitions [97, 98, 111, 115, 122, 130, 138]. Suppression of breast cancer xenografts by 74% was shown in mice [137]. In prostate cancer xenografts 40% reduction in growth was seen [112, 117] indicating that combinatorial approaches will be needed [112, 118]. Following this conclusion Ketola et al. found several compounds could potentiate disulfiram in reducing prostate cancer xenograft viability [104]. The vascular endothelial growth factor (VEGF) receptor kinase inhibitor sunitinib for example was synergistic with disulfiram [104].

Disulfiram also inactivates P-glycoprotein [139], a major drug exporter from the brain, and therefore a problem for us in delivering chemotherapeutic drugs to glioblastoma.

Antitumor efficacy specifically of temozolomide can be directly increased by disulfiram partially through inhibition of the DNA repair protein O6-methylguanine-DNA methyltransferase (MGMT) [130, 140]. MGMT is expressed at low levels in human brain. It normally functions to remove methyl groups from methylguanine that if left uncorrected would be read as adenosine (the normal G:C becomes an A:T) [141, 142]. MGMT transfers the temozolomide-generated methyl groups bound to O6-position of guanine directly to an active site cysteine (Cys145) in MGMT in a 1:1 stoichiometric reaction [143], permanently destroying the MGMT molecule that is then degraded after each single methylguanine demethylation [144]. Consistent with this, OS patients with MGMT-proficient tumors is somewhat shorter than those with lower expression of MGMT [145, 146].

Lesions that escape MGMT-mediated removal are targeted by mismatch repair (MMR). During DNA replication temozolomide-induced unrepaired methylguanine mispairs with thymine. MMR removes thymine from these methylG-T mispairs. As methylguanine is left unrepaired by MMR the methylguanine is again read as an adenosine and paired with thymidine. This becomes a futile repair cycle. This may result in single-strand DNA repair patches that block replication. In a subsequent round of replication, this eventually results in double-strand breaks that are potent activators of the apoptotic pathway [141, 142].

Inhibition of MGMT by O6-benzylguanine during temozolomide is undergoing clinical trials but frequent bone marrow toxicity is discouraging [147]. Due to its basic microenvironment MGMT's Cys145 has a low pKa of 4.8 [148]. Srivenugopal and his colleagues have found that disulfiram can form adducts with this Cys 145 at the active site of MGMT, thereby inactivating its methyltransferase function [144, 149]. Empirically disulfiram increases the cytotoxicity of another aklylating agent occasionaly used in glioblastoma treatment, BCNU (Paranjpe). From the foregoing, it is clear that the electrophilic drug disulfiram, a major component in the CUSP9 protocol, is expected to have multifaceted biochemical effects, all working in synergy to improve glioma treatment.

**Figure 4:**
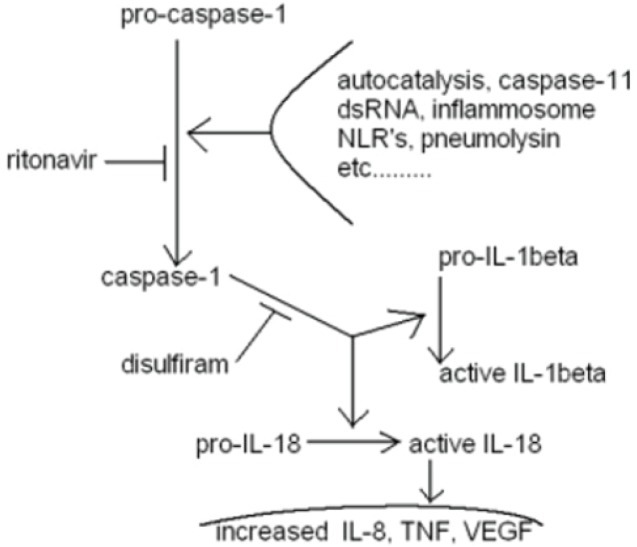
Diagram of another aspect of disulfiram + ritonavir of potential benefit to glioblastoma treatment effectiveness Caspase-1 is synonymous with ICE, interleukin-1 converting enzyme. The diagram lists ritonavir. It remains unproven if nelfinavir will function similarly, although every indication so far is that it will.

As highly reactive compounds, disulfiram and its first metabolite diethyldithiocarbamate react with many proteins in the cell, particularly by binding metals in enzymes' active sites [113-120]. This has made the determination of a primary mechanism of action in cancer treatment of disulfiram and its multiple metabolites difficult. Several non-exclusive mechanisms have been proposed based on demonstrated in vitro actions summarized below:

- inhibition of DNA methyltransferase [112],
- reduction of NF-kB activation [100-102, 111, 116, 133, 150],
- reduction of DNA replication [118],
- induction of oxidative stress [100, 117],
- induction of mitochondrial membrane permeabilization, cell cycle arrest, reduction of angiogenesis, invasion of cancer stem cells [100],
- proteosome inhibition [115-118, 137, 151], as inhibitor of superoxide dismutase with consequently increased intracellular reactive oxygen species [152],
- MGMT repair function inhibition [133, 144, 149]
- inhibition of interleukin-1 converting enzyme (ICE-1) as diagramed in Fig. 4 [153, 154].
- ALDH inhibition mediated decreases cancer stem cell function [121, 122, 126, 127, 130, 155]
- P-glycoprotein inactivation [139]

Which of these paths is important in disulfiram's clinical anti-cancer effect, which are primary and which secondary to some underlying primary effect, which are simple epiphenomena, are all unanswered questions. Recent dramatic data from the lab of H. Ishii in Osaka indicate that ALDH enzymatic function itself is important for stem cell resistance to cytotoxic chemotherapy [155]. ALDH therefore is not just a marker for stemness. It is a mediator of stemness and most importantly, a mediator we can block.

Concordant with the data of H. Ishii's group and particularly encouraging for our intended use in CUSP9, Schäfer et al. have recently identified ALDH 1A1 isoform as a major marker (and mediator of) glioblastoma resistance to temozolomide [123]. We have ample reason to include disulfiram. In many ways disulfiram would seem to be the ideal adjuvant addition to current cytotoxic chemotherapies of cancer, including glioblastoma.

Since some data on disulfiram as adjunct during cancer treatment has indicated a requirement for copper [97- 99, 101, 105, 116, 119, 122]. Copper gluconate is a 434 Da salt, widely available without prescription as a dietary supplement that we add to CUSP9 on that basis. This might not be necessary in that it is thought that plain disulfiram rapidly chelates copper after ingestion [96]. The United States' FDA has concluded that copper (cupric) gluconate is a category 1, Generally Regarded as Safe (GRAS) substance. In their words category 1 means that “There is no evidence in the available information on (substance) that demonstrates, or suggests reasonable grounds to suspect, a hazard to the public when they are used at levels that are now current or might reasonably be expected in the future.” [156].

#### II.2.5 nelfinavir

Nelfinavir is a 568 Da oral aspartic protease inhibitor in use for over ten years in the treatment of HIV. It is usually well tolerated, short term side effects are few. If gradual up titration is observed, loose stool without need for treatment was the only immediate side effect of note. In HIV-infected persons, years of treatment with HIV protease inhibitors may lead to metabolic disturbances, lipodystrophy, and insulin resistance [157].

**Figure 5:**
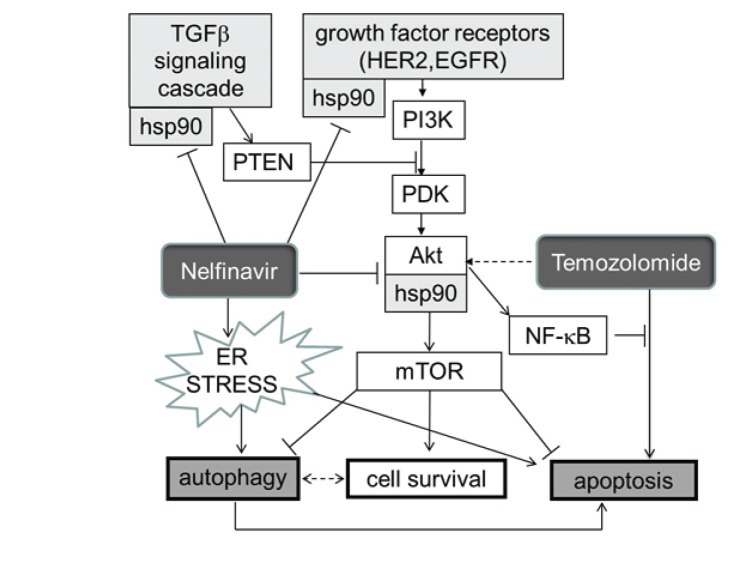
Schema indicating how HSP90, and hence its inhibition by nelfinavir (NFV) sit at several crossroads previously documented as crucial for glioblastoma growth Note that CUSP9 is expected to inhibit the compensatory survival-enhancing response to temozolomide by two paths. A) as indicated in this Figure via nelfinavir dampening of AKT function, and B] by the NFkB degradative actions of disulfiram. Maintainance of a good NFkB pool is one of the crucial elements in glioblastoma hardiness [314].

HSP90 is a dimeric ATP-binding chaperone protein that binds to and thereby conformationally stabilizes many signaling receptors and crucial intracellular proteins [158]. Many signaling receptors shown to be important in glioblastoma growth are such HSP90 stabilized proteins or protein complexes. Some of these are diagramed in Fig. 5. There are many other client proteins of HSP90 that are of potential importantance in glioblastoma growth. Nelfinavir, like the older, related, 721 Da protease inhibitor ritonavir, binds to and prevents or limits the chaperone function of HSP90 [159-161] with multiple beneficial cancer chemotherapy consequences, some of which are outlined below.

**Figure 6:**
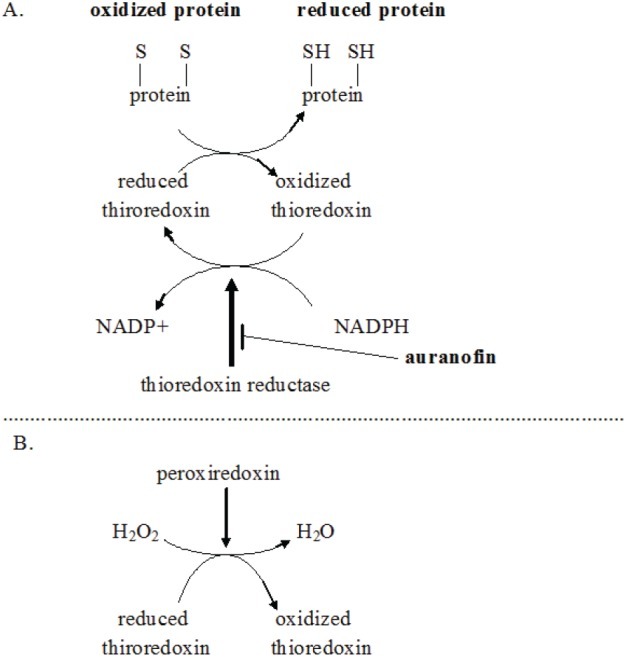
**A.** Schema showing auranofin shifting of intracellular redox towards an oxidizing state by diminishing regeneration of reduced thioredoxin. **B.** Schema showing related path by which thioredoxin reductase inhibition increases intracellular hydrogen peroxide (H_2_O_2_). Particular importance of NADPH regeneration from NADP+ in glioblastoma metabolism and growth is reviewed in ref. 313.

Wide interest in nelfinavir as an effective new anti-cancer drug [162-165], in particular in the adjuvant role in combination treatment, isreflected by the implementation of more than two dozen clinical studies with cancer patients as of the end of 2012 (www.clinicaltrials.gov) that are currently ongoing, including two studies with glioma patients (NCT00915694: nelfinavir plus temozolomide plus radiation; NCT01020292: nelfinavir plus temozolomide).

Increased endoplasmic reticulum (ER) stress [163, 167] and HSP90 function interference [161] are major paths by which nelfinavir is thought to exert an anti-cancer effect. Loci of nelfinavir's undermining of several core survival enhancing paths engaged by temozolomide can be seen diagrammed in Fig. 5.

Nelfinavir-mediated ER stress, probably caused by nelfinavir's inhibition of a yet unidentified ER associated aspartic proteinase involved in protein processing, leads to a massive accumulation of misfolded proteins within the ER lumen, triggering the pro-apoptotic pathway of the ER stress signaling cascade [163, 167, 168]. In glioma cells, nelfinavir-mediated ER stress was shown to up regulate pro-apoptotic proteins and to induce cleavage of caspase 4 in vitro and in a xenograft model [168].

Nelfinavir has not only been shown to be effective against a wide variety of different cancer cell lines at clinically relevant levels [169-171], but more importantly has also been shown to be similarly effective on ex vivo biopsy tissue from patients with primary and recurrent ovarian cancer [163]. This indicates a possible use of nelfinavir for relapsed cancer, independent of previously acquired chemoresistance. Particularly in myeloma cells can impaired proliferation and proteosome inhibition be seen after exposure to nelfinavir, with curiously enhanced inhibition to that provided by the marketed proteosome inhibitor bortezomib [171].

Experimental (not marketed) inhibitors other than nelfinavir or ritonavir e.g., derivatives of geldanamycin like 17-allylamino-17-demethoxygeldanamycin (17-AAG), are in active research programs as adjuncts to traditional current cancer cytotoxic chemotherapy. Trials of these drugs are based on strong pre-clinical evidence that HSP90 inhibition undermines several compensatory survival enhancing responses to temozolomide in glioblastoma [172], in other cancers [173] and in gliomas to other cytotoxic chemotherapeutic drugs [174-176].

Specific studies on temozolomide action in glioma have shown HSP90 inhibition (again mostly but not uniquely using 17-AAG) as a strong sensitizing maneuver to glioma cells otherwise resistant to temozolomide [174, 177] or irradiation [178]. Several novel [not marketed] HSP90 inhibitors have shown strong cytotoxicity against glioblastoma specifically against the stem cell sub-population [175, 176, 179, 180]. Empirical inhibition is seen of proliferation of glioblastoma cell lines U251, A172, and U373 during exposure to nelfinavir alone but without significant apoptosis induction [181]. Increased vulnerability in nelfinavir-exposed cells to apoptosis induction by other agents was however demonstrated [181].

Matrix metalloproteinase-2 (72 kDa, MMP-2, also termed gelatinase A) and MMP-9 (94 kDa, also termed gelatinase B) are proteolytic collagenases strongly associated with glioblastoma growth and invasion [159, 182, 183]. MMP-9 secretion is an HSP90 requiring event [184]. Note that three of CUSP9 drugs inhibit MMP-2, MMP-9- captopril, disulfiram, and nelfinavir, as referenced in the respective sections of this paper, and have previously been suggested as a mutually reinforcing trio of drugs well-suited as mutually reinforcing adjuvants in the treatment of glioblastoma [159].

Phosphatase and tensin homologue (PTEN) is a phosphatase that constitutively inhibits or functions to diminish AKT (protein kinase B, a serine/threonine kinase) signaling. PTEN is often malfunctioning in glioblastoma, resulting in AKT related constitutive overdrive. AKT is one of the many intracellular proteins requiring HSP90 chaperoning for optimal functioning. Experimental HSP90 inhibitors like 17-AAG [177] as well as nelfinavir and ritonavir inhibit AKT function [161, 185-190], partially reversing faulty PTEN function as commonly seen in, and forming one of the core growth enhancing abnormalities of, glioblastoma [191-193]. In 2006 Pore et al. showed decreased VEGF, hypoxia induced factor-1 (HIF-1), and AKT activation in U87 glioma cells and decreased angiogenesis into U87 seeded, subcutaneously placed Matrigel plugs in mice treated with oral nelfinavir [194, 195].

We therefore expect some of the growth drive consequent to PTEN loss common in glioblastoma [193] resulting overactivation of AKT to be partially reversed by nelfinavir.

Of the many proteins that use HSP90 for correct folding and function, one of the most important in glioblastoma pathophysiology is perhaps transforming growth factor-beta (TGF-beta) [196-199]. That TGF-beta sits at a crucial crossroads in glioblastoma's dysregulation as recently reviewed by Eyler and Rich makes HSP90 a particularly attractive target [30]. This crossroads position of HSP90 in this regard is also indicated in Fig. 5.

Benefit was clear when nelfinavir was added to standard chemoradiation in a recent study of unresectable non-small cell lung cancer [200]. Of particular note was that this benefit was achieved using the dose we suggest for CUSP9, 1250 mg p.o. twice daily, and that this gave little evidence of added side effect burden to patients [200].

A remarkable research of Xie et al. showed that “nelfinavir is able to inhibit multiple members of the protein kinase-like superfamily” in addition to lowering AKT activation. Perhaps most interesting of all was their observation and their accompanying conceptual leap, that weak inhibition of multiple growth enhancing kinases as provided by nelfinavir can result in significant anti-cancer activity, not necessarily by itself but by setting up the intracellular milieu to be less robust in coping with other insults like irradiation or traditional cytotoxic drugs [190], or in our case nelfinavir undermining survival paths that are engaged after exposure to temozolomide.

We expect nelfinavir to be well tolerated. In HIV studies only about 4% of nelfinavir treated patients switched to other drugs due to side effects [201] and in a recently reported clinical study of nelfinavir as cancer treatment adjuvant nelfinavir added little morbidity [200].

For treatment of HIV-infected persons, nelfinavir is more and more being replaced by ritonavir-boosted HIV protease inhibitors of the newer generation. Some of these HIV protease inhibitors, including lopinavir, atazanavir [168], and even ritonavir as a single agent [185,189, 202], have been shown to have similar anti-tumoral (but this does not imply identical) effects as nelfinavir. Since nelfinavir is the currently most investigated HIV drug for cancer treatment, this treatment plan will focus on the incorporation of nelfinavir but remains open for the inclusion or replacement by other HIV protease inhibitors in case that preclinical or clinical data indicate a superiority of other marketed HIV protease inhibitors in glioblastoma treatment.

### II.3 Published reports of increased OS with use but of uncertain significance and drugs with less robust theoretical support- captopril, sertraline, ketoconazole

#### II.3.1 sertraline

Sertraline is a 306 Da selective serotonin re-uptake inhibitor (SSRI) in common use over the last twenty years in the treatment of depressed mood [203]. It is usually well-tolerated and is effective in about half of patients started on it [203, 204].

By formal study of glioblastoma patients, an 18% rate of moderate to severe depression is seen post-resection [205] but other studies would suggest that the actual rate of depression-related signs and symptoms was considerably higher and that these significantly degraded quality of life in glioblastoma patients [206]. Family member interviews indicate that glioblastoma patients experience greater depression signs than the patients themselves report [207].

A recent study of 1,364 glioma patients showed a non-significant longer survival [“suggestive trend towards a beneficial association”] in those treated post-diagnosis with tricyclic antidepressants [208]. Not only don't we know if this data is a real effect or not (potential for propter hoc fallacy) but also we don't know that, if it were a real association (longer survival if treated with a tricyclic antidepressant) would this carry over to the modern SSRI's generally or sertraline specifically. However Caudill et al. at the Mayo Clinic reported also a non-significant longer survival in glioblastoma patients on an SSRI class antidepressant such as sertraline compared to those not on such…”Two-year survival in the cohort of patients taking an SSRI was 32% versus 17% in those who were not (P = 0.18).” [209]. No excess toxicity was noted in the SSRI treated patients [209].

Additive anti-proliferative effect in U87 glioblastoma cells was seen with temozolomide plus sertraline in vitro [210]. Down regulation of AKT with some reduced proliferation was seen in melanoma cells exposed to sertraline [211] but it was undetermined if this was related to serotonin reuptake inhibition or some other attribute of sertraline. So all above considerations taken together we consider the minimal risks of adding sertraline to be worth any gains that might accrue, were this data, particularly of slight increases in OS, to hold.

#### II.3.2 captopril

Angiotensin conversion enzyme inhibitors (ACEI) are a class of eminently well-tolerated drugs commonly used to treat hypertension, or congestive heart failure [212, 213]. ACEI inhibit the proteolytic cleavage of angiotensin I to angiotensin II. A related class of drugs, sartans, binds to and prevents the stimulation of the angiotensin II receptor.

In a study of 87 glioblastoma patients, those on ACEI or a sartan had lower need for dexamethasone [214]. This steroid-sparing use of ACEI alone holds potential to improve QOL, in that psychiatric [215, 216], metabolic [217], and immunosuppressive [218] morbidities secondary to dexamethasone use in glioblastoma are not trivial. The Carpentier et al. study's finding of a non-significant increase in OS in the ACE inhibited group was intriguing [ACEI use had “no effect on survival (16.2 vs. 17.9 months for the treated and the non-treated group, respectively, P = 0.77)”] [214]. Data like this not meeting statistical significance, as with the non-significant increase in OS noted in Caudil et al.'s study of SSRIs, can both a) be a real, important, and ultimately statistically significant difference that becomes evident on further study with larger populations, and b) allow important insights into pathophysiology of a given disease if the difference proves to be statistically significant even if not clinically meaningful to patients in itself.

Captopril is a 217 Da oral ACEI, the first such to enter clinical use [219]. Since then many other ACEI's have been approved yet captopril remains useful in treating hypertension and as remodeling inhibitor post-myocardial infarction [219]. Although hypotension is a theoretical risk, captopril has a history of safe use in uncomplicated hypertension. Orthostatic hypotension incidence remains low even in setting of captopril use in congestive failure and post-myocardial infarction [220].

In experimental systems, the antitumor effects of diverse ACE inhibitors and sartans show that as a class they inhibit cell proliferation and possess antiangiogenic, antimetastatic effects in multiple cancer models; hepatocellular [221], squamous cell [222], renal cell [222-224], gliomas [225, 226], bladder [227], ovarian [228], prostate [229], breast [230], colon [231], gastric [232]. On this basis as well we feel the ACEI effect of Carpentier et al. will prove to be validated as a slight but statistically significant benefit when greater numbers are studied.

Human glioblastoma cells express immunohistochemistry demonstrable renin and angiotensinogen mRNAs and proteins, as well as renin and angiotensin 1 or 2 receptors [233]. Fully 67% of human glioblastomas expressed angiotensin 1 receptors, 53% expressed angiotensin 2 receptors [225].

Losartan, a sartan class pharmaceutical angiotensin II receptor blocker, reduced the growth of C6 glioma in rats [234]. Glioma cell line T98G constitutively synthesizes MMP-2 and MMP-9. This is inhibited by captopril with an expected consequent inhibition of Matrigel invasion inhibition [235]. As human glioblastoma tissue produces prodigious amounts of both MMP-2 and MMP-9, collagenases strongly associated with glioblastoma growth and invasion [159, 182, 183] captopril was previously mentioned as an obvious therapeutic intervention for glioblastoma [159].

#### II.3.3 ketoconazole

Ketoconazole is a 531 Da broad spectrum anti-fungal drug, used in both topical and oral applications [236]. Several paths of importance in glioblastoma growth have good experimental documentation of inhibition by ketoconazole. To what extent ketoconazole congeners like miconazole or fluconazole share the discussed properties of ketoconazole is likewise unknown. Fluconazole penetration into brain tissue approaches 1:1 with blood levels but it might not possess the four attributes of ketoconazole outlined below.

Ketoconazole is a potent inhibitor of a) 5- lipoxygenase [237- 239], b) thromboxane synthase [240- 243], c) a drug efflux pump at the blood-brain barrier [244- 250], and d) has shown an empirical inhibition of cancer cell growth [238, 239].

In a study of long-term ketoconazole treatment of onychomycosis, 18% of people developed slight liver transaminase elevation, 3% developed transaminase elevations high enough to trigger stopping ketoconazole [251]. Reviews on the risks/benefits of ketoconazole and related anti-fungal drugs have concluded risks for ketoconazole use are low [252]. A total of about ten case studies have been published of fatal hepatitis during oral ketoconazole treatment [253].

In advanced prostate cancer patients on docetaxel given adjunctive ketoconazole at 200 mg p.o. three times daily this was well-tolerated, giving 1.24 microg/mL trough and 2.79 microg/mL peak plasma ketoconazole levels during steady-state [254].

##### a) Ketoconazole & 5-lipoxygenase

Arachidonate 5-lipoxygenase (arachidonate:oxygen 5-oxidoreductase, EC 1.13.11.34) is the rate-limiting enzyme in leukotriene synthesis. A proliferation enhancing role in glioblastoma for 5-lipoxygenase generated leukotrienes was first suggested in 1998 when inhibition of 5-lipoxygenase was shown to inhibit proliferation in U-373 glioma cell line [255]. dl-nordihydroguaiaretic acid (“Nordy”) is a phytoderived 5-lipoxygenase inhibitor active in inhibiting CD133+ related clonogenicity of glioma cell lines [256]. Glioblastoma biopsy material studied by immunohistochemistry for 5-lipoxygenase shows heavy staining [257]. Two experimental 5-lipoxygenase inhibitors inhibited proliferation of the A172 glioma cell line [257], results confirmed and extended a year later by a different group [258]. A heavy granular staining for 5-lipoxygenase is seen in glioblastoma [and grade 3 astrocytomas] not seen in the even staining of normal neurons and glia [259].

Much of the peritumoral edema seen during glioblastoma is generated by 5-lipoxygenase mediated leukotriene synthesis [260- 262]. Human glioblastomas are prodigious producers of leukotrienes [263] the consequence from which derives many of the decrements in QOL and increases in tumor growth vigor. Glioblastoma patients' urinary leukotriene excretion decreases by 79% post-resection [263] indicating that it was the tumor itself that was generating excess leukotrienes. Exposure to experimental 5-lipoxygenase inhibitors inhibited in vitro glioma cell growth [264, 265]. Increased 5-lipoxygenase in glioblastoma was first noted in 2003 [266], then confirmed in a 2006 immunohistochemistry study showing staining for 5-lipoxygenase where heavier staining correlated with shorter OS [267]. The immunochemistry study of Ishii in 2009 again confirmed this and furthermore showed that experimental 5-lipoxygenase inhibitors inhibit proliferation of A172 glioblastoma cell line [257].

Already in 1989 increased leukotrienes in areas of glioblastoma peritumoral edema were documented [262]. On this basis we can expect diminished peritumoral edema with consequently increased QOL in ketoconazole treated glioblastoma patients as well as, if the data of Ishii et al. and the other indices of anti-glioblastoma effects mentioned hold, longer OS.

##### b) Ketoconazole & thromboxane synthase

Robust thromboxane A2 content of, and synthetic ability by, human glioblastomas was shown as early as 1987 [268]. Thromboxane A2 receptors are widely expressed on normal tissues and on various cancers, including human glioblastoma cells [269- 271]. Thromboxane synthase catalyzes the formation of thromboxane A2 from prostaglandin H2.

Already in 1998, unregulated gene expression with corresponding increase in mRNA for thromboxane synthase was noted in glioblastoma cells selected in vitro for enhanced migration [272]. An experimental thromboxane synthase inhibitor [(furegrelate) inhibited glioblastoma cell line growth in vitro and growing in nude mice and most importantly sensitized these cells to he alikylating chemotherapy drug BCNU [273]. This confirmed and extended earlier work showing that thromboxane synthase inhibition diminished in vitro migration and sensitized glioblastoma cell lines to killing by irradiation [274].

Following documentation of increased thromboxane synthase gene and protein expression in the sub-set of glioblastoma cells with a more migratory phenotype compared to the slower moving majority population [277] an in vitro study of exposure to the experimental thromboxane synthase inhibitor furegrelate resulted in caspase activation, DNA fragmentation, and apoptotic death [275].

Thromboxane synthase as a growth facilitating element also in many non-glioma cancers- and the beneficial potential benefit from its inhibition- is an active area of current research [276]. We intend to use the thromboxane synthase inhibitor we already have on the market- ketoconazole.

##### c) Ketoconazole as efflux pump inhibitor

Volume sensitive osmolyte efflux was blocked by ketoconazole [244].

P-glycoprotein efflux of docetaxel at blood brain barrier was inhibited by ketoconazole [245]. We have evidence for ketoconazole inhibiting BBB efflux of phenobarbital [246] and ritonavir [160, 248]. Ketoconazole inhibits the human breast cancer resistance protein (BCRP) [250], and P-glycoprotein efflux pump [247- 249]. Its occasional use in psychiatric practice is based on a) the salutary consequence of ketoconazole's blunting high cortisol excursions [277] and b) increase in brain tissue of certain psychotropic medicines by virtue of its efflux pump inhibition [278].

## IV CUSP9 PHARMACOLOGY

### IV.1 Introduction

The introduction of a therapy with ten-drugs requires a special assessment of safety to limit the risk of adverse drug reactions. The greater the number of drugs, the closer should be the monitoring. We judge a minimum of twice weekly un-hurried meetings with gradual one-by-one addition of drugs and slow up titration will be best for safety. Drug-drug interactions represent a common event and these should be prevented with an adequate protocol of dosing that requires an early assessment of potential interplays between the scheduled medicines. To assess potential interactions each drug of CUSP9 was combined to the others as shown in the grid of Fig. 3. Each pair of drugs was evaluated for the available evidence of interactions using as source of information the Risk Control Plans (RCP) of the reference medicinal product [279], and by a specific research using MEDLINE and Embase.

### IV.2 Pharmacokinetic interactions

The literature review has provided the evidence that four potential pharmacokinetic interactions may occur for the group of drugs scheduled in the protocol: ketoconazole + aprepitant, nelfinavir + aprepitant, artesunate + ketoconazole and artesunate + nelfinavir.

#### IV.2.1 Ketoconazole + aprepitant and nelfinavir + aprepitant

CYP3A4 is the major enzyme involved in the metabolism of aprepitant [280]. Ketoconazole is well-known as one of the most potent inhibitors known of CYP3A4. In vitro studies showed that ketoconazole may inhibit the 98% of aprepitant metabolism, with a marked increase in its bioavailability (5-fold increase of AUC, 3-fold increase of half-life) [280, 281]. Nelfinavir inhibits CYP3A4 [282, 283]. No study has directly tested the effect of nelfinavir on aprepitant levels or metabolism. It is therefore difficult to quantify the pharmacokinetic result of this potential interaction. In terms of clinical relevance, if used in standard doses, the combination ketoconazole-nelfinavir-aprepitant may result in very high levels of aprepitant. In the previously mentioned antidepressant article 300 mg. aprepitant per day (twice the dose suggested for CUSP9) gave no side effects different from placebo [36, 49].

#### IV.2.2 Ketoconazole + artesunate and nelfinavir + artesunate

As mentioned above, ketoconazole and nelfinavir are both potent inhibitors of CYP3A4. CYP3A4 is also involved in the metabolism of artesunate [284]. Nelfinavir and ketoconazole can be expected to increase artesunate levels. As a consequence, the possibility of artesunate dose-related adverse reactions is enhanced, among which the most relevant would be QT prolongation [285]. A QTc of >o.42 will exclude artesunate from CUSP9.

### IV.3 Pharmacodynamic interactions

The literature review did not provide direct evidence of specific pharmacodynamic interaction among the drugs included in the protocol. However, considering the main categories of adverse drug reaction for each drug, a potential hepatotoxic effect is expected for ketoconazole [286], nelfinavir [287] and temozolomide [288]. Patients exposed to the combination of these three drugs should be monitored by at least monthly assessments of liver functions.

A further potential pharmacodynamic interaction would be that between auranofin and artesunate. The use of artesunate is contraindicated in patients receiving aurothioglucose since both drugs have been associated with the development of blood dyscrasias [289]. Although a specific contraindication with auranofin is not reported, we should expect risk for similar interaction with auranofin + artesunate, warranting frequent blood counts when these are given.

### IV.4 Felicitous drug-drug interactions

IV.4. 1. Protection against artesunate cytotoxicity is afforded by intracellular ROS reducing agents [58- 60], important elements of which of which are inhibited by auranofin [81,82] and disulfiram [100, 117, 152].

IV.4. 2. Auranofin does double duty. First as thioredoxin reductase inhibitor [290, 291] then as cathepsin B inhibitor [79, 80, 84, 94].

IV.4. 3. Note that three CUSP9 drugs inhibit MMP-2 & MMP-9- captopril, disulfiram, and nelfinavir [159, 292]

IV.4. 4. Two drugs of CUSP9 have inhibitory activity at relevant drug efflux pumps, disulfiram [108, 109, 139], and ketoconazole [245- 250].

IV.4. 5. Both disulfiram [98, 100, 121- 124] and nelfinavir and other HSP90 inhibitors [294- 296] have shown specificity in inhibiting cancer stem cell function.

IV.4. 6. Both artesunate [297] and nelfinavir [194, 195] lower both VEGF and HIF-1 activity.

IV.4. 7. Interference with AKT function has been noted after exposure to sertraline [211] and nelfinavir [55, 65].

IV.4.8. Disulfiram and ritonavir acting together inhibit ICE-1, lowering IL-1 beta growth drive to glioblastomas [153, 154], also diagramed in Fig. 4.

## V RISK REDUCTION, RISK ASSESSMENT, AND PARTIAL CUSP'S

### V.1 Safety features built into CUSP9

Underpinning the entire CUSP9 treatment process and a crucial component of CUSP9 are the safety features that must be in place for a new many-drug protocol like this. Sudden onset drug-drug interactions can usually be prevented by considering, as we have done, researching literature and history of drugs under consideration and their individual pharmacologic attributes. Specific safety requirements of CUSP9 are therefore:

#### V.1.1 Frequent meetings

Onset of drug-drug interactions is usually gradual. Early recognition of a negative interaction is enhanced by slow up titration. Unhurried careful weekly meetings are required for CUSP9 to function safely. We will have better chances to catch incipient unexpected interactions quickly this way.

#### V.1.2 Addition of one drug at a time

To minimize unforeseen drug-drug interactions with a complicated but necessary regime like CUSP9, a single drug only is added at each meeting. The next drug is added only when it has been established that that latest added drug has not generated a problem. The one exception to this rule will be copper gluconate that will be given with each disulfiram or not given if disulfiram is not tolerated.

#### V.1.3 Slow up titration

Drugs will be added at the low end of their dose range and slowly up titrated if meeting with the patient, and lab study if needed, establish that the last dose has been well tolerated.

#### V.1.4 Frequent lab monitoring

Weekly check of EKG, liver, kidney, and bone marrow function will be a minimum.

V.1.5

Patients will have a 24 hour phone contact with their monitoring physician and instructions to call if there is any deterioration in any domain.

#### V.1.6 Exclusions

A lengthy and detailed exclusion criteria list will help safety. Among many other exclusions, herbal preparations, nutritional supplements, or over-the-counter medicines or other ancillary medicines will not be allowed with 3 exceptions: 1) Hypertension controlled on any combination of ACEI or sartans will be permitted, in which case captopril is omitted from CUSP9, and 2) non-insulin requiring diabetes well controlled on metformin will be allowed. The use of grapefruit juice or extracts will be discouraged because of its CYP3A4 inhibiting effect. 3) Hydromorphone will be allowed for pain control.

#### V.1.7 Partial CUSP's

If an untoward reaction or side effect is seen with the addition of a particular drug, that drug will be dropped from that patient's treatment protocol and the addition process will continue as planned minus that drug, after restitution to the state of well-being established prior to addition of the offending drug. No further additions will be given if such restitution does not occur.

### V.2 Our safety assessment

Using our clinical experience with these drugs combined with published data, to help others understand the safety structure better we have stratified the relative risk of the ten drugs as temozolomide >auranofin > ketoconazole > nelfinavir >artesunate > sertraline > captopril > disulfiram > aprepitant > copper gluconate.

For heuristic reasons and as options for patients and physicians who are timorous we offer a suggestion to consider partial CUSP's in four risk category combinations.

#### V.2.1 Low risk combination

Given at the low end of our target dosing range, aprepitant 80 mg twice daily, sertraline 50 mg twice daily, captopril 25 mg twice daily, disulfiram 250 mg twice daily, copper gluconate 2 mg twice daily.

#### V.2.2 Medium risk combination

artesunate or auranofin, nelfinavir, ketoconazole in addition to the low risk drug combination.

#### V.2.3 Higher risk combination

temozolomide, combination auranofin and artesunate in addition to drugs of the low and medium group, the full CUSP9 given at our suggested target doses:

artesunate 50 mg p.o. twice daily
aprepitant 80 mg p.o. twice daily
sertraline 50 mg p.o. twice daily
captopril 50 mg p.o. twice daily
auranofin 3 mg p.o. twice daily
nelfinavir 1250 mg p.o. twice daily
temozolomide 25 mg/M^2^ p.o. twice daily
disulfiram 250 mg p.o. twice daily
copper (cupric) gluconate 2 mg p.o. twice daily
ketoconazole 200 mg p.o. twice daily

## VI A CUSP9 SUMMARY

The use of old drugs for new indications, coined “repurposing” is a realistic concept to accelerate therapy development for many cancers, previously formulated by many others [for example 25, 113, 120, 299, 300]. We herein add our voice and suggest a specific, coordinated old drug mix- CUSP9- as a multipronged coordinated attempt to augment current treatment of recurrent glioblastoma. In parallel with bacteriology practice we believe that a combination strategy such as CUSP9 is less likely to allow development of chemotherapeutic resistance as usually occurs in cancer treatment and specifically limits clinical efficacy of temozolomide in glioblastoma.

We, The International Initiative for Accelerated Improvement of Glioblastoma Care, heartily invite suggestions, comments, or proposals for additions or deletions, or further improvement of the CUSP9 protocol, or its translational use in other cancer treatments. Indeed allowing wide debate, critical thinking, and feedback with intent to improve CUSP9 was a central motive for publishing this protocol. Comments to either of the corresponding authors will be sent to all co-authors for discussion and evaluation.

We are not the first (nor was Virchow when he discussed this in mid-1800's) to suggest a connection between inflammation pathways and cancer, and to consider blocking these as a cancer treatment modality [301- 305]. The term “inflammation” is too imprecise to be useful in discussing the relationship between the malignant clone/cells and the rest of the body [301, 302]. CUSP9 aims to inhibit elements of inflammation that enhance glioblastoma growth.

There are many open questions. To what degree do nelfinavir and ritonavir share attributes? To what degree does ketoconazole increase CSF levels of nelfinavir as it does for ritonavir? Will BBB opening maneuvers be required for any of the CUSP9 drugs? Will any of the drugs work against any of the others in ways we don't foresee? To what extent are associations with angiotensin inhibition and longer OS, or SSRI use and longer OS, simple propter hoc fallacies or real meaningful or causal elements?

Two of our CUSP9 drugs- disulfiram and ritonavir/nelfinavir have a potential synergy in inhibiting a growth path used in glioblastoma that resembles closely an IL-1beta converting enzyme (ICE-1, also termed caspase-1) and IL-18 mediated inflammation paths in some cases of acute pancreatitis [153], diagramed in Fig. 4. “Reducing IL-1beta and IL-18 production by inhibition of ICE-1 is one promising strategy…” [303] in cancer treatment [294, 301- 303, 306] that was outlined already in 2001 by Randle's group in München. IL-18 and IL-1beta are both well documented growth facilitating elements in glioblastoma [306, 307]. We take up this thread by using disulfiram and nelfinavir to do exactly that- ICE inhibition with disulfiram and nelfinavir to inhibit IL-1 and IL-18 activation, details of which are pharmacologically explained in ref. 153 and depicted in Fig.4. We intend to thereby re-re-wire the pathogenically re-wired, amplifying feedback loop between HIF-1 and IL-1 common in cancers generally as described by Kaluz and Van Meir [307].

Aspects of our approach are similar to that being implimented in pediatric glioblastoma by J. Wolff et al. [308] at Tufts University where relapse post-primary treatment is selected in part by morphometric and immunohistochemical data that show which markers an individual tumor bears, then searching for already-marketed drugs that may block that receptor system or marker. This can lead to use of non-cytotoxic drugs not traditionally thought of as cancer chemotherapeutic drugs- as in CUSP9.

As referenced throughout our paper, CUSP9 is weighted towards interference with glioblastoma stem cell function, a particularly fruitful sub-population to target, offering higher reward yet having similar risks as targeting the tumor cell population as a whole [309, 310]. Glioblastoma stem cells have a complex interaction with their surrounding brain parenchyma, stroma and extracellular matrix [311]. There is a bidirectional relationship between these two compartements (surrounding brain and the glioblastoma cell) [311, 312] with which CUSP9 aims to block, as discussed throught this paper.

Both that >99% of patients will experience progression post-primary treatment and the short median OS of patients with glioblastoma warrant taking the measured and manageable risks of CUSP9. The 22 reports of failed studies of new treatments using variations on traditional cancer therapeutic published in 2012 [1, 3-23] also justify our excursion into conceptually new treatment approaches as here in CUSP9. CUSP9 complexity and requirement for large clinician time commitment per treated patient may be unavoidable if we are to make a dent in this tough disease.

So as in Preamble- ecce turtur. ipse proficit tantum con collum foras.
